# Direct vs Video Laryngoscopy for Difficult Airway Patients in the Emergency Department: A National Emergency Airway Registry Study

**DOI:** 10.5811/westjem.2022.6.55551

**Published:** 2022-08-19

**Authors:** Brandon T. Ruderman, Martina Mali, Amy H. Kaji, Robert Kilgo, Susan Watts, Radosveta Wells, Alexander T. Limkakeng, Joseph B. Borawski, Andrea E. Fantegrossi, Ron M. Walls, Calvin A. Brown

**Affiliations:** * Duke University Medical Center, Department of Emergency Medicine, Durham, North Carolina; † Texas Tech University Health Sciences Center, Department of Emergency Medicine, El Paso, Texas; ‡ Harbor-UCLA Medical Center, Department of Emergency Medicine, Torrance, California; § Brigham and Women’s Hospital, Department of Emergency Medicine, Boston, Massachusetts

## Abstract

**Introduction:**

Previous studies suggest improved intubation success using video laryngoscopy (VL) vs direct laryngoscopy (DL), yet recent randomized trials have not shown clear benefit of one method over the other. These studies, however, have generally excluded difficult airways and rapid sequence intubation. In this study we looked to compare first-pass success (FPS) rates between VL and DL in adult emergency department (ED) patients with difficult airways.

**Methods:**

We conducted a secondary analysis of prospectively collected observational data in the National Emergency Airway Registry (NEAR) (January 2016–December 2018). Variables included demographics, indications, methods, medications, devices, difficult airway characteristics, success, and adverse events. We included adult ED patients intubated with VL or DL who had difficult airways identified by gestalt or anatomic predictors. We stratified VL by hyperangulated (HAVL) vs standard geometry VL (SGVL). The primary outcome was FPS, and the secondary outcome was comparison of adverse event rates between groups. Data analyses included descriptive statistics with cluster-adjusted 95% confidence intervals (CI).

**Results:**

Of 18,123 total intubations, 12,853 had a predicted or identified anatomically difficult airway. The FPS for difficult airways was 89.1% (95% CI 85.9–92.3) with VL and 77.7% (95% CI 75.7–79.7) with DL (P <0.00001). The FPS rates were similar between VL subtypes for all difficult airway characteristics except airways with blood or vomit, where SGVL FPS (87.3%; 95% CI 85.8–88.8) was slightly better than HAVL FPS (82.4%; 95% CI, 80.3–84.4). Adverse event rates were similar except for esophageal intubations and vomiting, which were both less common in VL than DL. Esophageal intubations occurred in 0.4% (95% CI 0.1–0.7) of VL attempts and 1.5% (95% CI 1.1–1.9) of DL attempts. Vomiting occurred in 0.6% (95% CI 0.5–0.7) of VL attempts and 1.4% (95% CI 0.9–1.9) of DL attempts.

**Conclusion:**

Analysis of the NEAR database demonstrates higher first-pass success with VL compared to DL in patients with predicted or anatomically difficult airways, and reduced rate of esophageal intubations and vomiting.

## INTRODUCTION

### Background

Direct laryngoscopy (DL) has been the historical standard for airway management in the emergency department (ED); however, the use of video laryngoscopy (VL) has steadily risen over the past decade. As of 2012, about 55% of ED intubations were performed using DL, compared with 39% using VL.^1^ Prospective, single-center observational studies have demonstrated that VL improves glottic exposure and intubation success in ED and intensive care unit patients.^2^–^6^ Furthermore, multiple studies have shown that VL use among emergency medicine residents has been associated with fewer adverse events, including esophageal intubations.^2^–^6^ In spite of these promising results concerning VL, recent randomized trials in critical care patients and one meta-analysis of randomized trials with various patient types have not shown a clear benefit of one intubation method over the other. However, these studies do not fully represent ED populations since many studies excluded difficult airways and rapid sequence intubation or included primarily less experienced internal medicine trainees as intubators.^7^–^10^

### Importance

One of the proposed advantages of VL is an absolute reduction in the number of failed intubations in patients with difficult airways, as suggested by multiple systematic reviews.^11^–^12^ Difficult airways are more likely to require multiple attempts and are associated with an increased rate of complications and peri-intubation adverse events including esophageal intubation, airway trauma, and hypoxia.^13^–^17^ Video laryngoscopy has become increasingly used in ED intubations, and variations in VL design (hyperangulated vs standard geometry blade shape) can affect the mechanics of intubation and may improve first-pass success (FPS).^18^

### Goals of This Investigation

Our primary goal in this study was to measure the rates of FPS comparing VL vs DL intubations in adult ED patients who had an anticipated or identified anatomically difficult airway. We also sought to answer the question of whether VL design (hyperangulated vs standard geometry) influenced FPS in these patients. Our secondary goal was to determine whether there were differences in peri-intubation adverse events between these two intubation methods.

## METHODS

### Study Design and Setting

This was a retrospective analysis of data from the National Emergency Airway Registry (NEAR), a prospective, multicenter registry of ED intubations from 25 academic and community hospitals. Site investigators at each participating center were responsible for ensuring that data entry was completed for at least 90% of intubations performed in the ED and that the ED intubations were confirmed by comparison with institutional coding data or respiratory department capture of ED intubation procedures. All participating sites obtained approval from their local institutional review boards to conduct and participate in the study prior to data collection.

**Population Health Research Capsule**

What do we already know about this issue?

*Video laryngoscopy is the most common intubation method used in academic emergency departments, yet its benefit in patients with difficult airways remains unknown*.

What was the research question?

*Is video laryngoscopy associated with higher first-pass success than direct laryngoscopy in difficult airways?*

What was the major finding of the study?

*Video laryngoscopy had higher rates of first-pass success for difficult airways than direct laryngoscopy (89.1% [95% CI 85.9–92.3] vs 77.7% [95% CI 75.7–79.7]), respectively*.

How does this improve population health?

*This study supports using video laryngoscopy for difficult airways, which may lead to improved patient outcomes with fewer failed intubation attempts and adverse events*.

### Selection of Participants

All adult patients with an attempted ED intubation from January 1, 2016–December 31, 2018 were eligible for inclusion in the study. We excluded pediatric patients (defined as <15 years of age), patients who had an initial attempt with a device besides DL or VL (such as fiberoptic intubations), and those who were missing data on attempt, success, device, or patient age.

### Measurements

Intubating clinicians entered all registry data into a secure, web-based data collection form requiring institution-specific login credentials and passwords (StudyTRAX; version 3.47.0011 (ScienceTRAX, Macon, GA). Variables collected included patient demographics, body habitus, estimated weight, pre-intubation hemodynamics, methods of preoxygenation, initial intubator gestalt of airway difficulty (ie, physician anticipation that the intubation could be challenging), observable difficult airway characteristics (eg, mouth opening, Mallampati score, neck mobility, presence of airway obstruction, etc), intubation position and device, medications and doses, operator characteristics, first-pass intubation success or failure, adverse events, and patient disposition. After data upload, study investigators reviewed all data, using quality assurance algorithms to identify and correct data entry errors. The study coordinator performed active compliance monitoring to ensure that a 90% reporting threshold was maintained registry-wide by cross-referencing captured intubations reported by each site with their online entries. All data is reported in accordance with the Strengthening the Reporting of Observational Studies in Epidemiology (STROBE) statement (www.strobe-statement.org).

### Outcomes

The primary outcome measure was FPS among adult patients with difficult airways stratified by DL and VL. We defined a difficult airway as any intubation that was either anticipated to be difficult by the operator (physician gestalt) or had at least one of the following recorded difficult airway characteristics: greater than normal body habitus (obese or morbidly obese); reduced neck mobility; Mallampati score greater than two; reduced mouth opening; thyromental distance less than two fingers; airway obstruction present; facial trauma; or blood or vomit in the airway. Further, we performed a subgroup analysis comparing FPS rates by the type of video laryngoscope used: standard geometry VL (SGVL) and hyperangulated VL (HAVL). The SGVL devices included the C-MAC (Karl Storz SE & Co., Tuttlingen, Germany) and McGrath MAC (Medtronic, Minneapolis, MN), while HAVL devices included the GlideScope (Verathon Inc, Bothell WA), King Vision (Ambu, Inc, Ballerup, Denmark), and C-MAC D-blade. An intubation attempt was defined as insertion of the device into the mouth past the alveolar ridge regardless of whether the attempt was successful or not.

Our secondary outcome was the rate of adverse events as specified by the NEAR data collection form. We reported rates for cardiac arrest (loss of pulses during or immediately after intubation), esophageal intubation, hypoxia (oxygen saturation <90% during intubation when starting at a value >90% or a decrease in oxygen saturation by 10% if starting at a value <90%), and vomiting with aspiration. We chose to highlight these adverse events as they were among the most commonly considered to be directly influenced by FPS. Additional recorded adverse events were extremely rare and, therefore, reported together as “any adverse event.” These included dental trauma, direct airway injury, epistaxis, hypotension (systolic blood pressure <100 millimeters of mercury), iatrogenic bleeding, lip laceration, laryngoscope failure, laryngospasm, mainstem intubation, pharyngeal laceration, pneumothorax, or tracheal tube cuff failure.

### Statistical Analysis

We exported all study data from StudyTRAX to SAS v 9.4 (SAS Institute, Inc, Cary, NC) for statistical analysis. To account for within-site correlations, we performed a cluster analysis using the *proc surveyfreq* function in SAS. We first described cluster-adjusted binomial distributions of FPS, stratified by DL, VL, HAVL, and SGVL. We then described the differences between these cohorts based upon previously described predictors that affect FPS and rates of adverse events.^13^ We also reported the exact binomial distributions for adverse events for DL, VL, HAVL, and SGVL.

## RESULTS

**Figure 1:**
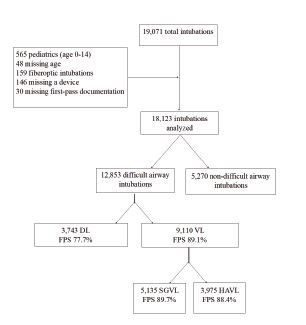
Flowsheet of intubations included and analyzed during the study period. Note that 948 intubations were excluded; neither were 5,270 intubations included in the final analysis as they did not meet criteria for a difficult airway. *DL*, direct laryngoscopy; *VL*, video laryngoscopy; *FPS*, first-pass success; *HAVL*, hyperangulated video laryngoscopy; *SGVL*, standard geometry video laryngoscopy.

During the 36-month study period, 19,071 intubations were recorded in the registry. After applying the above exclusion criteria, 18,123 remained. Of these, 12,853 (71%) were classified as difficult airways and included in the final analysis (Figure 1). Direct laryngoscopy was performed on 3,743 (29.1%) of these, and VL on 9,110 (70.9%). Patient and intubation characteristics are shown in Table 1. The overall FPS rate of VL was significantly higher than that of DL (89.1% vs 77.7%, *P* <0.00001). Approximately 72% of included patients were under 65 years old, about two-thirds were male, and 70.2% were intubated for a medical indication. Nearly half (46.3%) of the intubations included obese or morbidly obese patients. The most common method of intubation used for medically indicated difficult airways was SGVL, whereas for traumatic indications HAVL was most commonly used. Of the difficult intubations, 46.9% were anticipated to be difficult based on gestalt alone, indicating that the remainder of difficult intubations were classified as such due to an anatomic predictor.

First-pass success was significantly higher for VL than for DL for all difficult airway characteristics with the exception of “airway obstruction present” (Table 2). Table 2 compares FPS rates for VL and DL among all difficult airway characteristics included in the NEAR survey. For airways that were anticipated to be difficult by the operator, FPS was significantly higher for VL than DL by 13.7% (85.0% vs 71.3%). Stratifying VL by blade shape revealed a similar FPS rate for HAVL and SGVL (88.4% vs 89.7%) in difficult airway patients (Table 3). Interestingly, “blood or vomit in the airway” was the only difficult airway characteristic for which there was a statistically significant difference in FPS between HAVL (82.4%; 95% confidence interval [CI] 80.3–84.4) and SGVL (87.3%; 95% CI 85.8–88.8).

**Figure 2:**
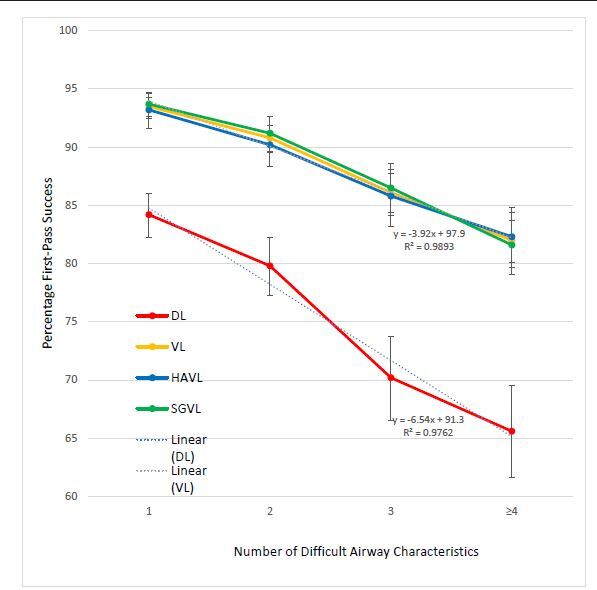
First-pass success rates for direct laryngoscopy (DL), video laryngoscopy (VL), hyperangulated VL (HAVL), and standard geometry VL (SGVL) for patients with multiple difficult airway characteristics. The percentage first-pass success is shown for DL, VL, HAVL, and SGVL for increasing numbers of distinct difficult airway characteristics. 95% confidence intervals are shown as error bars. Linear regression trendlines and their respective equations are shown for DL and VL. *DL*, direct laryngoscopy; *VL*, video laryngoscopy; *FPS*, first-pass success; *HAVL*, hyperangulated video laryngoscopy; *SGVL*, standard geometry video laryngoscopy.

In Table 4, we show a comparison of FPS between DL, VL, HAVL, and SGVL as increasing numbers of difficult airway characteristics are added. The FPS gradually decreases for each method of intubation as the number of difficult airway characteristics increases (Figure 2). The FPS for VL overall, as well as HAVL and SGVL individually, remains higher than the FPS for DL regardless of the number of difficult airway characteristics. When linear trendlines are added to DL and VL, the slope representing the decrease in percentage FPS as additional characteristics are added is greater for DL than for VL (−6.54, R^2^ 0.98 vs −3.92, R^2^ 0.99). Furthermore, there does not appear to be any significant difference in the overall FPS rates between HAVL and SGVL for any number of characteristics.

For our secondary outcome, hypoxia was the most common individual adverse event, observed at a rate of 8.0% (95% CI 6.3–9.7) for all difficult airways (Table 5). When taken as a whole, there was no observable difference in the rates of adverse events between VL and DL (12.9% vs 13.5%). However, the rates of both vomiting and esophageal intubation were significantly lower among the difficult airways intubated with VL than those with DL. Esophageal intubation was observed in 1.5% (95% CI 1.1–1.9) of difficult airways intubated with DL compared to 0.4% (95% CI 0.1–0.7) for those intubated with VL. Similarly, the DL rate of vomiting was 1.4% (95% CI 0.9–1.9) and the VL rate was 0.6% (95% CI 0.5–0.7). There were also no observable differences in adverse event rates when comparing HAVL and SGVL for difficult airways.

## DISCUSSION

Although the use of VL has risen steadily over the past few years, the advantages and disadvantages of VL and DL continue to be debated.^1^,^5^,^19^,^20^ A 2018 meta-analysis of five randomized controlled trials with data from 1,250 patients found no significant difference in the first-pass or overall intubation success rates for VL and DL.^21^ However, many of the included trials systematically excluded patients with difficult airways, who could potentially benefit the most from the use of VL.^7^,^22^,^23^ Direct laryngoscopy requires alignment of the oral, laryngeal, and pharyngeal axes to visualize the glottis, whereas VL, depending on blade shape, either does not require the same degree of alignment (SGVL) or no alignment at all (HAVL). The SGVL uses much of the same laryngoscopic technique as DL whereas HAVL requires a distinct technique both for glottic visualization and tube delivery. The HAVL is often suggested to be useful for patients with reduced neck mobility or when optimal patient positioning cannot be achieved, as it requires less “lifting force”; however, indirect tube delivery via a video screen can make tracheal tube placement challenging. We did not find an overall difference in FPS between these two subtypes, suggesting that, in general, operators are equally likely to succeed in difficult airways with VL regardless of blade shape and technique differences. The HAVL was, however, the most common subtype used for difficult airways with a traumatic indication, likely due to its benefit in patients with cervical collars and reduced neck mobility.

To our knowledge, our study is the largest to date that investigates the differences between DL and VL specifically for difficult airways. We found that the overall FPS was significantly higher for VL than DL by about 11.4% among patients with at least one difficult airway characteristic, and by about 13.7% for patients with anticipated difficult airways. Furthermore, the FPS for airways anticipated to be difficult was in general similar to that of anatomic predictors of difficult airways, with the exception of “airway obstruction present.” This suggests that physician gestalt for airways in the NEAR database is likely a reliable stand-alone predictor of a difficult airway, at least in terms of estimating FPS. “Airway obstruction present” was also the only characteristic that did not show a statistically significant difference in FPS between DL and VL for difficult airways. The exact reason for this is unclear but may be partially due to the small number of included airways with this characteristic, although there does appear to be a trend toward higher FPS for VL. In a few specific situations, mechanical obstructions in airways are easier to maneuver around with direct visualization rather than using a screen. Significant obstructing upper airway pathology may also equally limit endotracheal tube insertion for all device types, reducing the power to detect a difference.

First-pass success using VL was similar whether using HAVL (88.4%) or SGVL (89.7%). These results suggest that the primary advantage that VL offers in difficult airways is improved glottic visualization and that blade shape and indirect tube placement do not significantly alter FPS rate. The FPS for SGVL was slightly higher for patients with “blood or vomit in the airway” compared to HAVL (87.3% vs 82.4%). One possible explanation for this observed difference may be that the standard geometry blades allow for more effective suction through movement and management of the tongue, whereas the angle of HAVL blades limits suctioning of the oropharynx.

An increase in the number of individual difficult airway characteristics results in an expected linear decrease in the FPS, with the lowest success rate being 65.6% for DL for airways with four or more characteristics. Interestingly, the rate of decline in FPS appears to be faster for DL than both subtypes of VL as well. When comparing airways with four or more difficult airway characteristics to those with only one, VL FPS decreases by 11.5% (93.5% to 82.0%) while DL FPS decreases by 18.6% (84.2% to 65.6%). The benefits of VL may, therefore, be additive for increasingly difficult airways. Another interesting observation is that there did not appear to be any additive benefit for HAVL compared to SGVL for increasingly difficult airways.

The rate of adverse events for all difficult airways was 13.1%, which was similar between VL and DL. The choice of hyperangulated or standard geometry VL also did not appear to result in any difference in the rate of adverse events. The five most common adverse events among all difficult airways were hypoxia, hypotension, cardiac arrest, vomiting, and esophageal intubation. The remaining adverse events listed in the NEAR survey were extremely rare. We chose not to report the rate of hypotension alone, as this was likely affected more by medication selection and underlying patient physiology and pathology rather than the type of blade used.

We did observe a small but significant difference in the rate of esophageal intubations among DL first-pass intubations compared to VL. This result is consistent with the findings from multiple other studies that demonstrated a reduction in esophageal intubation rates with the use of VL.^5^,^21^ Fortunately, this was still a relatively rare event for difficult airways, occurring in only 0.7% of all first attempts. Esophageal intubations can be corrected on subsequent attempts if recognized, but we were unable to determine whether there was any association with other serious adverse events such as hypoxia or cardiac arrest due the small sample size of esophageal intubations. Future studies with larger numbers of esophageal intubations may help clarify whether there exists any correlation with increased risk of other adverse events. Vomiting was also more than twice as common among difficult DL intubations compared to VL (1.4% vs 0.6%). The reason for this difference is not entirely known but may be related to less lifting force (and secondary opening of the upper esophageal sphincter) as well as less direct pharyngeal and vagal stimulation with VL than with DL.

Our findings are very relevant for clinical practice in emergency medicine, as repeated intubation attempts have been shown to be associated with an increase in peri-intubation adverse events; thus, FPS should be the primary goal for all emergent intubations.^2^,^15^,^17^ While the effect of VL on FPS for routine airways is less clear and still debated, our results in this large cohort are consistent with smaller studies and suggest that VL should be the device of choice for airway management in the difficult airway in the ED.^7^,^8^,^14^,^24^

## LIMITATIONS

Our study has several important limitations. Although our data suggests an association between the use of VL and higher first-pass intubation success rates for difficult airways, we cannot determine a causal relationship due to the observational nature of this study and the inherent risk of confounding bias. Selection bias may also have occurred, as while we can report the type of laryngoscope used, it is not known why an operator may have selected it for a particular patient. The majority of our data also comes from academic EDs and, therefore, the rate of VL use and expected outcomes may not be generalizable to all settings, particularly more rural locations. In the most resource-limited settings and field environments, VL may not be feasible without the appropriate infrastructure or even an electrical grid. Additionally, preference for VL in academic institutions may contribute to underdeveloped DL skills among trainees and worse performance when confronted with difficult airways. Non-academic EDs with different patient and clinician populations and laryngoscope comfort may observe different results.

We did not compare operator preferences between academic and rural settings, as the proportion of data in the NEAR registry from non-academic settings represents too small a sample size to draw conclusions. Future studies of FPS rates among difficult airways in rural and resource-limited settings would serve as a useful comparison to our data. These studies would also better allow educators to teach intubation methods with the highest likelihood of first-pass success depending on the learner’s practice setting.

Although this study’s findings indicate that VL improves FPS in patients with difficult airways, we cannot demonstrate whether the clinician’s predictions of difficulty were correct. Further research is needed to help physicians develop their ability to predict difficult airways and choose the best approach.^25^–^26^ Our finding that FPS for airways anticipated to be difficult was similar to those with anatomic predictors suggests, however, that physician gestalt may be a relatively accurate predictor. We chose our list of difficult airway characteristics based on frequently studied attributes (mouth opening, thyromental distance, Mallampati score, obstruction, neck mobility, etc).^13^–^14^ However, other difficult airway characteristics and confounders may not be included. Additionally, all the characteristics were based on a subjective assessment by the operator. Over time this subjective assessment through experience becomes an operator’s gestalt. The “LEMON” rule has often been applied in preoperative airway assessments and has been modified in previous studies to “LEON” as the Mallampati score is often not performed in the ED setting.^25^–^27^

Finally, there is potential for self-reporting bias, as failure at first attempt intubations could have potentially influenced how the operator entered the airway characteristics into the survey. The data also may have not been entered by the operator immediately after the intubation attempt due to the emergent nature of intubation and, therefore, could have been subject to recall bias. Although there is potential for self-reporting bias with selective reporting of intubation attempts and failures, we believe that the site requirement of 90% compliance with data entry should have minimized this potential bias.

## CONCLUSION

We observed a higher overall first-pass success rate when using VL compared to DL in adult ED patients with characteristics of an anticipated or anatomically difficult airway. This advantage that VL offers appears to be additive as airways become increasingly difficult. The FPS rates between hyperangulated video laryngoscopy and standard geometry video laryngoscopy were similar for all difficult airway characteristics with the exception of “blood or vomit in the airway,” in which SGVL seemed to offer a slight advantage. Overall, the adverse event rates were similar between VL and DL with only the rates of esophageal intubation and vomiting being significantly lower with VL than DL. There was no difference in adverse event rates between SGVL and HAVL. Our data suggests that video laryngoscopy, either hyperangulated or standard geometry, should in general be the primary device used for difficult airway management in the ED. Future studies in resource-limited settings may help determine whether these benefits remain true when operators have less experience and training with VL.

## Figures and Tables

**Table 1:** Patient characteristics and use of DL, VL, HAVL, and SGVL* for first-pass intubation attempts among those with difficult airway characteristics.

	Total N = 12,853	DL n = 3,743 (29.1, 17.2–41.1)	VL n = 9,110 (70.9, 58.9–82.8)	HAVL n = 3,975 (30.9, 20.4–41.5)	SGVL n = 5,135 (40.0, 20.2–59.7)
Age 15–65	9,235 (71.9, 66.9–76.8)	2,698 (72.1, 65.7–78.5)	6,537 (71.8, 66.3–77.2)	2,801 (70.4, 65.3–75.7)	3,736 (72.8, 65.6–79.9)
Age > 65	3,618 (28.1, 23.2–33.1)	1,045 (27.9, 21.5–34.3)	2,573 (28.2, 22.8–33.7)	1,174 (29.5, 24.3–34.7)	1,399 (27.2, 20.1–34.4)
Male	8,500 (66.2, 63.9–68.4)	2,463 (65.8, 62.1–69.6)	6,037 (66.3, 64.0–68.6)	2,563 (66.5, 63.1–69.9)	3,394 (66.1, 63.7–68.5)
Female	4,349 (33.8, 31.6–36.1)	1,279 (34.3, 30.4–37.9)	3,070 (33.7, 31.4–36.0)	1,332 (33.5, 30.1–36.9)	1,738 (33.9. 31.5–36.3)
Habitus (very thin)	371 (2.9, 2.2–3.6)	132 (3.5, 2.7–4.4)	239 (2.6, 2.0–3.3)	116 (2.9, 2.2–3.7)	123 (2.4, 1.6–3.2)
Habitus (thin)	1,519 (11.9, 9.5–14.2)	468 (12.5, 9.9–15.1)	1,051 (11.6, 8.9–14.3)	499 (12.6, 10.6–14.6)	552 (10.8, 7.0–14.5)
Habitus (normal)	4,991 (38.9, 35.6–42.2)	1,423 (38.1, 33.9–42.3)	3,568 (39.3, 35.9–42.7)	1,518 (38.3, 33.4–43.2)	2,050 (40.0, 36.5–43.5)
Habitus (obese)	4,952 (38.6, 36.1–41.1)	1,490 (39.9, 35.8–44.0)	3,462 (38.1, 35.4–40.8)	1,409 (35.6, 32.9–38.3)	2,053 (40.0, 37.3–42.8)
Habitus (morbidly obese)	989 (7.7, 6.3–9.1)	222 (5.9, 4.7–7.2)	767 (8.4, 6.8–10.2)	421 (10.6, 8.4–12.8)	346 (4.7, 3.8–5.5)
Medical indication	9,029 (70.2, 63.2–77.3)	2752 (73.4, 65.2–81.9)	6,358 (69.8, 62.3–77.3)	2,512 (63.2, 52.7–73.7)	3,846 (74.9, 68.7–81.1)
Traumatic indication	3,743 (29.1, 22.2–36.1)	991 (26.5, 18.1–34.8)	2,752 (30.2, 22.7–37.7)	1463 (36.8, 26.3–47.3)	1,289 (25.1, 18.9–31.3)
Anticipated to be difficult	5,987 (46.9, 42.3–51.5)	1,695 (45.3, 41.1–49.6)	4,292 (47.5, 41.8–53.3)	2002 (50.4, 46.5–54.4)	2,’290 (45.3, 37.6–52.9)
First-pass success	11,028 (85.8, 82.3–89.3)	2,908 (77.7, 75.7–79.7)	8,120 (89.1, 85.9–92.3)	3,513 (88.4, 86.9–89.9)	4607 (89.7, 84.7–94.8)

Data are reported as N (%, 95% confidence interval).

* *DL*, direct laryngoscopy; *VL*, video laryngoscopy; *HAVL*, hyperangulated video laryngoscopy; *SGVL*, standard geometry video laryngoscopy.

**Table 2:** Comparative first-pass success rates of direct laryngoscopy and video laryngoscopy for each difficult airway characteristic.

	DL N = 3,743 (29.1, 28.3–29.9)	DL FPS n = 2,908 (77.7, 75.7–79.7)	VL N = 9,110 (70.9, 70.1–71.7)	VL FPS n = 8,120 (89.1, 85.9–92.3)
Anticipated to be difficult	1,695 (45.3, 41.1–49.6)	1,208 (71.3, 69.0–73.4)	4,292 (47.5, 41.8–53.3)	3,647 (85.0, 83.9–86.0)
Habitus > normal	1,712 (45.7, 40.9–50.6)	1,312 (76.6, 74.6–78.6)	4,229 (46.4, 43.3–49.6)	3,751 (88.7, 87.7–89.6)
Reduced neck mobility	1,146 (30.6, 25.4–35.8)	883 (77.1, 74.5–79.5)	3,758 (41.5, 34.6–48.3)	3,340 (88.9, 87.8–89.9)
Mallampati score > 2	668 (51.3, 45.6–56.9)	499 (74.7, 71.2–78.0)	1,647 (50.0, 41.6–58.4)	1,429 (86.8, 85.0–88.4)
Mouth opening < normal	683 (35.2, 26.6–43.7)	479 (70.1, 66.5–73.5)	1,963 (41.8, 32.8–50.8)	1,661 (84.6, 82.9–86.2)
Thyromental distance < 2 fingers	59 (4.4, 2.3–6.5)	36 (61.0, 47.4–73.5)	187 (5.1, 3.9–6.3)	156 (83.4, 77.3–88.4)
Airway obstruction present	175 (4.7, 3.0–6.4)	118 (67.4, 60.5–74.3)	498 (5.5, 4.7–6.3)	376 (75.5, 71.5–79.2)
Facial trauma	483 (12.9, 7.6–18.2)	368 (76.2, 72.1–79.9)	1,480 (16.3, 12.9–19.7)	1304 (88.1, 86.3–89.7)
Blood or vomit in the airway	1,503 (40.2, 32.5–47.9)	1089 (72.5, 70.1–74.7)	3,273 (36.1, 33.8–38.5)	2,792 (85.3, 84.0–86.5)

Data are reported as N (%, 95% confidence interval).

*DL*, direct laryngoscopy; *VL*, video laryngoscopy; *FPS*, first-pass success.

**Table 3:** Comparative first-pass success rates of hyperangulated video laryngoscopy (VL) and standard geometry VL for each difficult airway characteristic.

	HAVL N = 3,975 (43.6, 42.6–44.7)	HAVL FPS n = 3,513 (88.4, 86.9–89.9)	SGVL N = 5,135 (56.4, 55.3–57.4)	SGVL FPS n = 4,607 (89.7, 84.7–94.8)
Anticipated to be difficult	2,002 (50.4, 48.8–51.9)	1,699 (84.9, 83.2–86.4)	2,290 (44.6, 43.2–46.0)	1,948 (85.1, 83.5–86.5)
Habitus > normal	1,830 (46.0, 44.5–47.7)	1,595 (87.2, 85.5–88.7)	2,399 (46.7, 45.3–48.1)	2,156 (89.9, 88.6–91.0)
Reduced neck mobility	2,024 (50.9, 49.4–52.5)	1,801 (89.0, 87.5–90.3)	1,734 (33.8, 32.5–35.1)	1,539 (88.8, 87.2–90.2)
Mallampati Score > 2	748 (18.9, 17.6–20.1)	651 (87.0, 84.4–89.4)	899 (17.5, 16.5–18.6)	778 (86.5, 84.1–88.7)
Mouth opening < normal	956 (24.1, 22.7–25.4)	818 (85.6, 83.2–87.7)	1,007 (19.6, 18.5–20.7)	843 (83.7, 81.3–85.9)
Thyromental distance < 2 fingers	99 (2.5, 2.0–3.0)	84 (84.9, 76.2–91.2)	88 (1.7, 1.4–2.1)	72 (81.8, 72.2–89.2)
Airway obstruction present	223 (5.6, 4.9–6.4)	163 (73.1, 66.8–78.8)	275 (5.4, 4.8–6.0)	213 (77.5, 72.1–82.3)
Facial trauma	732 (18.4, 17.2–19.7)	643 (87.8, 85.3–90.1)	748 (14.6, 13.6–15.6)	661 (88.4, 85.9–90.6)
Blood or vomit in the airway	1,348 (33.9, 32.4–35.0)	1,111 (82.4, 80.3–84.4)	1,925 (37.5, 36.2–38.8)	1,681 (87.3, 85.8–88.8)

Data is reported as N (%, 95% confidence interval).

*HAVL*, hyperangulated video laryngoscopy; *SGVL*, standard geometry video laryngoscopy; *FPS*, first-pass success.

**Table 4:** First-pass success rates for airways with increasing number of difficult airway characteristics.

	Number of difficult airway characteristics			

	1	2	3	≥4
DL FPS	1,229/1,460 (84.2,82.2–86.0)	850/1,065 (79.8, 77.3–82.2)	455/648 (70.2, 66.5–73.7)	374/570 (65.6, 61.6–69.5)
VL FPS	2,965/3,171 (93.5, 92.6–94.3)	2,212/2,437 (90.8, 89.5–91.9	1,485/1,724 (86.1, 84.4–87.7)	1,458/1,778 (82.0, 80.1–83.8)
HAVL FPS	1,096/1,176 (93.2, 91.6–94.6)	969/1,074 (90.2, 88.3–91.9	687/801 (85.8, 83.2–88.1)	761/924 (82.4, 79.7–84.8)
SGVL FPS	1,869/1,995 (93.7, 92.5–94.7)	1,243/1,363 (91.2, 89.6–92.6)	798/923 (86.5, 84.1–88.6)	697/854 (81.6, 78.9–84.2)

Data is reported as ratios (%, 95% confidence interval).

*DL*, direct laryngoscopy; *VL*, video laryngoscopy; *FPS*, first-pass success; *HAVL*, hyperangulated video laryngoscopy; *SGVL*, standard geometry video laryngoscopy.

**Table 5:** Adverse event rates during first-pass intubation attempts for DL, VL, HAVL, and SGVL* among difficult airways.

	Total N = 12,853	DL n = 3,743	VL n = 9,110	HAVL n = 3,975	SGVL n = 5,135
Cardiac arrest	125 (1.0, 0.7–1.2)	42 (1.1, 0.8–1.5)	83 (0.9, 0.6–1.2)	38 (1.0, 0.6–1.3)	45 (0.9, 0.6–1.2)
Esophageal intubation	94 (0.7, 0.4–1.0)	58 (1.5, 1.1–1.9)	36 (0.4, 0.1–0.7)	18 (0.5, 0.2–0.8)	18 (0.4, 0.1–0.7)
Hypoxia	1,027 (8.0, 6.3–9.7)	293 (7.8, 5.9–9.7)	734 (8.1, 6.2–9.9)	325 (8.2, 6.0–10.4)	409 (8.0, 5.8–10.1)
Vomiting	108, (0.8, 0.7–1.0)	52 (1.4, 0.9–1.9)	56 (0.6, 0.5–0.7)	26 (0.7, 0.5–0.8)	30 (0.6, 0.4–0.8)
Any adverse event	1,676 (13.1, 10.9–15.3)	504 (13.5, 11.2–15.8)	1,172 (12.9, 10.3–15.5)	547 (13.8, 11.5–16.1)	625 (12.2, 9.0–15.5)

Data is reported as N (%, 95% confidence interval).

* *DL*, direct laryngoscopy; *VL*, video laryngoscopy; *HAVL*, hyperangulated video laryngoscopy; *SGVL*, standard geometry video laryngoscopy
